# Extending healthy ageing: nutrient sensitive pathway and centenarian population

**DOI:** 10.1186/1742-4933-9-9

**Published:** 2012-04-23

**Authors:** Sergio Davinelli, D Craig Willcox, Giovanni Scapagnini

**Affiliations:** 1 Department of Health Sciences, University of Molise, Campobasso, Italy; 2 Department of Human Welfare, Okinawa International University, Ginowan, Japan

## Abstract

Ageing is a challenge for any living organism and human longevity is a complex phenotype. With increasing life expectancy, maintaining long-term health, functionality and well-being during ageing has become an essential goal. To increase our understanding of how ageing works, it may be advantageous to analyze the phenotype of centenarians, perhaps one of the best examples of successful ageing. Healthy ageing involves the interaction between genes, the environment, and lifestyle factors, particularly diet. Besides evaluating specific gene-environment interactions in relation to exceptional longevity, it is important to focus attention on modifiable lifestyle factors such as diet and nutrition to achieve extension of health span. Furthermore, a better understanding of human longevity may assist in the design of strategies to extend the duration of optimal human health. In this article we briefly discuss relevant topics on ageing and longevity with particular focus on dietary patterns of centenarians and nutrient-sensing pathways that have a pivotal role in the regulation of life span. Finally, we also discuss the potential role of Nrf2 system in the pro-ageing signaling emphasizing its phytohormetic activation.

## Introduction

Ageing is an irreversible process associated with numerous physiological alterations across multiple organ systems. Molecular studies in model organisms have identified several longevity genes and pathways which can extend the lifespan. Although many data are available from these animal models, in humans the situation is much more complex. Certainly human ageing is due to interactions between genetic and epigenetic factors but in addition to the genetic background, successful or unsuccessful ageing is also determined by environmental factors associated with social structure, culture and lifestyle. A fascinating, rapidly emerging concept in the biomedical sciences which may help establish a novel and innovative intellectual framework in biomedical research is the 'positive biology paradigm' [1]. Rather than making disease the central focus of researchers' efforts, positive biology seeks to understand the causes of positive phenotypes and which biological mechanisms would explain health and well-being. For instance, a better understanding of exemplars of human exceptional longevity could be a goal of positive biology and could create real benefits for those who are more vulnerable to disease and disability. Life expectancy for humans has more than doubled in the last two centuries and in some European countries it is estimated that by 2050 the proportion of persons older than 60 will rise from 20% to almost 40% [2] and the number of centenarians will be nearly 3.2 million world-wide [3]. Therefore, even though scientists have elucidated many biological ageing processes providing new strategies that may help to slow the rate of ageing in humans, it is imperative to emphasize research on healthy ageing in order to reduce frailty and disability associated with the "normal" ageing process. Resistance against cellular stress and environmental insults can help promote a more successful ageing process that results in a longer and healthier lifespan. Despite the fact that genetic, nutritional and pharmacological interventions have been identified as potential means to slow ageing and extend lifespan in lower organisms [4], caloric restriction (CR) appears to be the only common way to increase lifespan in all species [5]. Nutrient sensors modulate lifespan extensions that occur in response to different environmental and physiological signals. Nutrient-sensing pathways are essential to the ageing process because several nutrients can activate different pathways directly or indirectly. Many of the genes that act as key regulators of lifespan also have known functions in nutrient sensing, and thus are called "nutrient-sensing longevity genes". Some examples of nutrient-sensing pathways involved in the longevity response are the kinase target of rapamycin (TOR) [6], AMP kinase (AMPK) [7], sirtuins [8] and insulin and insulin/insulin-like growth factor (IGF-1) signaling [9]. Among the processes and experiential factors that guide successful ageing trajectories, nutrition has been receiving much attention as a modifiable lifestyle factor leading to healthy ageing. Literature concerning the heterogeneity in dietary and nutritional status of centenarians seems to indicate that there is not any particular dietary pattern that promotes exceptional longevity. Although different nutritional compounds have been analyzed in studies of health ageing and longevity [10], it is crucial to understand how specific nutritional components and dietary patterns may affect health and longevity. To date the main dietary intervention that may retard the ageing process is CR and a rare human example could be the Okinawan population in Japan. Okinawans appear to have undergone a mild form of prolonged CR for decades that could have contributed to a lower risk of mortality [11]. A deep knowledge of the mechanisms underlying differences among centenarians from various countries would be beneficial, especially elucidating the contribution of country-specific dietary patterns. Here we will focus on specific nutritional patterns of centenarians located throughout the world considering the role of nutrient-sensing pathways in mediating the longevity response and beneficial effects. Finally, considering that CR is a mild stress that actives cytoprotective mechanisms, we discuss the potential role of Nrf2 protective cell-signaling pathway in CR induced longevity.

### Nutritional patterns of centenarians: nature vs. nurture

A challenge in the area of healthy ageing is to identify dietary patterns, in addition to specific dietary components, that offer protection against age-related diseases. Dietary patterns are defined mainly for assessing eating behavior and to relate the food intake to disease or health outcomes [12]. Although severe disabilities in persons older than 60 seem to be declining, the prevalence of chronic disease, particularly of those diseases linked to diet and lifestyle, appears to be increasing [13]. Healthy centenarians are ‘expert survivors’ with important lessons to share, in particular regarding the most modifiable lifestyle factor: the diet. However, it is necessary to emphasize that several lines of evidence suggest that the genetic contribution to a healthy life span in populations with exceptional longevity may be greater than that seen in the general population [14,15]. A recent study on a cohort of 477 Ashkenazi Jewish centenarians with exceptional longevity reported that centenarians may possess additional longevity genes that help to buffer them against the harmful effects of an unhealthy lifestyle [16]. Nonetheless, even though studies of dietary patterns are intrinsically complex, several reports have showed that specific dietary patterns are potentially associated with longevity [17,18]. The diets of 5 populations with extraordinarily high longevity have been recently described and labeled "Blue Zones". [19]. Populations of Okinawa, Japan; Sardinia, Italy; Loma Linda, California; the Nicoya Peninsula, Costa Rica and Ikaria, Greece seem to have a high prevalence of centenarians and a preferential attitude toward a plant based-diet. Interestingly, in 2003 Shimizu et al. investigated the dietary practices of 104 centenarians who lived in the Tokyo metropolitan area and reported that a dietary pattern based on dairy products was associated with increased survival [20]. Traditional Okinawan diets provide about 90% of calories from carbohydrates but in vegetable form [21] therefore are low in calories but nutritionally dense, particularly with regard to vitamins, minerals, and phytonutrients [18]. Okinawans also have a very high intake of phytochemicals in the diet. All plants contain these natural compounds and the elders seem to have significantly lower levels of lipid peroxidation and suffer less free-radical-induced damage. For many individuals, the cognitive changes that occur with ageing are affected by micronutrient intake. Recently, a pilot study compared circulating levels of micronutrients among cognitively healthy volunteers aged 85 years and older in Okinawa and Oregon. The Okinawan elders used fewer vitamin supplements but had similar levels of vitamin B12 and α-tocopherol, compared with Oregonian elders. Thus the components leading to healthy cognitive ageing might include a variety of patterns that include a healthy diet, high physical activity, and social engagement [22]. Additionally, cognitive function, daily activity, and residential status, have been reported to affect nutritional intake of centenarians [23,24]. The traditional Mediterranean diet provides about 40% of calories from fat, mostly monounsaturated and polyunsaturated fat [21]. The benefits of a Mediterranean diet are well known but a specific region in the Mediterranean island of Sardinia ('Blue Zone') is characterized by exceptional male longevity. Noteworthy, an analysis in the 377 Sardinian municipalities provided evidence that dietary variables are not significantly correlated with male extreme longevity. In particular, it was revealed that a lower caloric intake is not related with a superior level of longevity but rather factors affecting energy expenditure are important in explaining extreme longevity [25]. These findings seem to indicate that in this area CR had an impact, although minor on exceptional longevity. In Loma Linda, California, there is a community of Seventh Day Adventists which, according to several studies, live longer than the rest of the population. Interestingly, their vegetarian diet is thought to be the most likely cause of their extraordinary longevity. Specific dietary factors that may be involved in their outstanding health include a high intake of fruit, vegetables, and nuts [26-28]. The Nicoya Peninsula region in Costa Rica has been reported to be an exceptional longevity area where healthy centenarians live surrounded by a solid support network of friends and family [29]. Although the possible role played by the dietary regimen in Nicoya region in relation to extreme longevity has not yet been investigated, the diet includes garden vegetables, an abundance of fruit (orange, mango, papaya), squash, beans, rice and corn. The water is also particularly high in minerals such as magnesium and calcium [19]. This area has also reported one of the lowest middle-age mortality rates in the world. A 60-year -old has more than a fourfold better chance of making it to the age of 90 years than a 60-year-old in North America [30]. Finally, it was reported that people in Ikaria Island, Greece, have also one of the highest life expectancies in the world. Ikarians are three times more likely to reach the age of 90 years than in the U.S. [19]. In this community, scientific evidence shows protective health benefits from long-term adherence to the Mediterranean food culture revealing that this diet has a cardioprotective effect and is able to reduce the prevalence of hyperuricaemia in elderly individuals [31]. The above mentioned regions have been labeled as 'Blue Zones' and while scientists try to validate the veracity and variety of associated causes of this exceptional longevity, it is advisable to follow a diet rich in fruits, vegetables, legumes and whole grains but reduced in saturated fat.

### 'Nutrient sensors' that modulate ageing

Biochemical pathways capable of 'sensing' the availability of nutrients maintain energy homeostasis both at the cell and at the whole organism levels [32]. Multiple nutrient signaling pathways have been connected to lifespan regulation. Although an extremely low-calorie diet is the most effective intervention known to extend lifespan in many species, from yeast to primates [33], we will focus on nutrient-sensing pathways that have been shown to influence ageing in humans. The full understanding of dietary intake and composition to obtain health benefits and pro-longevity effects is a realistic goal for biogerontology and drugs that promote human longevity targeting nutrient-sensing pathways require further study. Moreover, it has been observed that natural genetic variants in nutrient-sensing pathways are associated with increased human life span [34]. In recent years, some of these pathways identified in worms or mice have been shown to have human homologs, in particular the IGF-1 pathway. IGF-1 activity is essential in all animals, and surprisingly, altered IGF-1 signaling pathways have been shown to confer an increase in susceptibility to longevity, including human longevity. Recent studies have demonstrated a significant association between mutations in genes involved in the IGF-1 pathway and extension of human life span. Indeed mutations known to impair IGF-1 receptor function have been shown to be overrepresented in a cohort of Ashkenazi Jewish centenarians suggesting that centenarians may harbor rare genetic variations in genes encoding components of the IGF-1 pathway [35]. Polymorphic variants of genes which are involved in IGF-1 signaling have also been linked to longevity in a Japanese cohort of 122 semi-supercentenarians (105 years old and older) [36]. The FOXO transcription factor FOXO3, part of the IGF-1 pathway, is essential for CR effects and it has been demonstrated that polymorphisms in FOXO3 are associated with human longevity in several cohorts. Specifically, building upon previous findings in animal models, Willcox et al. (2008) identified a strong association between FOXO3A and human longevity in Japanese-Americans from Hawaii [37]. Subsequently, Pawlikowska et al. reported common variants in both FOXO3A and AKT1, to be associated with longer lifespan in three independent Caucasian cohorts [38]. Anselmi et al. also validated the association between FOXO3A polymorphisms and extreme longevity in males from the southern Italian Centenarian Study [39]. Moreover, the key role of FOXO3 in human longevity has been further confirmed in German and Chinese centenarians [40,41]. However, despite the recognition of FOXO3 as a "master gene" in ageing the actual functional variant remains unidentified. Recently, Donlon et al. sequenced the coding region in their long-lived Japanese-American population demonstrating that of 38 published variants the vast majority remained unconfirmed, indicating that coding variants may not be key players [42]. Enhanced resources for fine-mapping this region are necessary. The TOR (target of rapamycin) pathway is an evolutionarily conserved nutrient-sensing pathway which has been implicated in the regulation of life span and in the response to stress, nutrients and growth factors [43]. As Blagosklonny argued, ageing may not necessarily be driven by damage, but, in contrast, lead- to damage and this process is driven in part by mTOR (mammalian target of rapamycin) [44]. However, to date human data have been scarce and the details of how mTOR exerts lifespan control and anti-ageing effects are still not fully understood. Notably, it was recently demonstrated that rapamycin reverses the phenotype of cells obtained from patients with Hutchinson-Gilford progeria syndrome, a lethal genetic disorder that mimics rapid ageing [45]. Taking into account that mTOR signaling is a major nutrient-sensing pathway with effects on ageing, the inhibition of this signaling pathway may be similar to what is seen in dietary restriction and, as discussed earlier, is known to extend the lifespan of diverse organisms [5]. Maintenance of mitochondrial activity is also an emerging topic in the field of ageing research and many reports indicate that an effective mitochondrial fitness is essential for healthy ageing [46]. AMPK is a nutrient and energy sensor that might be involved in the regulation of life span and in the mediation of the beneficial effects of CR. Although this hypothesis is largely unexplored, especially in mammals, it seem likely that the activation of AMPK may have an impact on the activity of FOXO, sirtuin, and the mTOR pathways, which have been linked to CR and to the promotion of a healthy longevity [47].

### Hormetic phytochemicals and Nrf2- signaling pathway in healthy ageing

**Figure 1:**
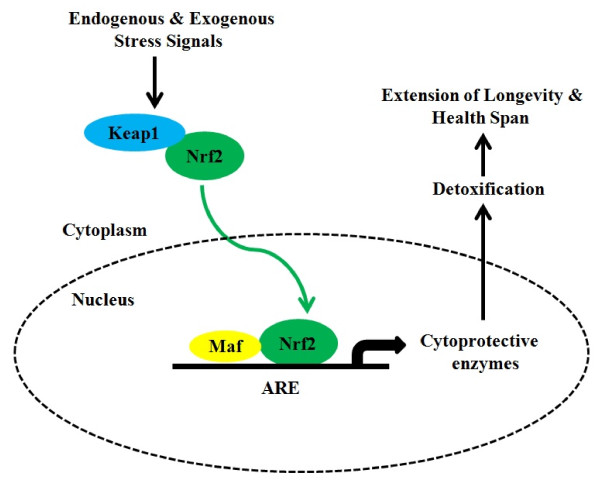
**General scheme for the induction of Nrf2-signaling pathway**. The antioxidant response element (ARE) in the promoter region of select genes allows the coordinated up-regulation of antioxidant and detoxifying enzymes in response to dietary phytochemicals. This up-regulation is mediated through nuclear factor (erythroid-derived 2)-like 2 (Nrf2) that may be activated by endogenous and exogenous molecules or stressful conditions. These agents disrupt the association between Nrf2 and Keap1 with subsequent nuclear translocation of Nrf2. In the cell nucleus Nrf2 interacts with small MAF protein, forming a heterodimer that binds to the ARE sequence in the promoter region and up-regulates transcription of many genes encoding detoxifying enzymes. We speculate that this signaling pathway is constitutively upregulated in long-lived individuals providing extension of longevity and health span.

There is a huge volume of data on the beneficial effects of plant-derived extracts to retard ageing and age-associated diseases. Many phytochemicals are synthesized to increase the fitness of the plant by allowing it to interact with its environment, including herbivorous pathogens, and insects. Some well-known phytochemicals may induce in humans beneficial stress responses and with low-dose exposures, they can trigger a cellular stress response and subsequently induce adaptive stress resistance, also called hormesis [48]. Stress resistance involves several molecular adaptations and induces many of the nutrient-sensing longevity pathways discussed above. The role of hormesis in ageing has already been examined by Rattan [49] but also its relevance to explain the anti-ageing and life-extending actions of CR in long-lived species [50]. We propose that the anti-ageing responses induced by phytochemicals are caused by phytohormetic stress resistance involving the activation of Nrf2 signaling. There is a substantial amount of research supporting oxidative stress as one of the main causes of ageing. In contrast there are few longevity models that have been created to evaluate enhanced anti-oxidative mechanisms. Nrf2 is a central regulator of the adaptive response to oxidative stress but very few studies have investigated the role of Nrf2 in the modulation of ageing and longevity. The Nrf2-signaling pathway has been extensively reviewed elsewhere [51,52] as well as its hormetic function from an evolutionary perspective [53]. It is important to delineate the link between oxidative stress, cellular resistance and the rate of ageing. Since Nrf2 regulates the main cytoprotective responses, this pathway may substantially contribute to the determination of healthspan and extension of longevity (Figure 1). Moreover, many phytochemicals (e.g. polyphenols, flavonoids, terpenoids, etc.) are major ingredients present in fruits, vegetables, and spices and have been shown to have protective effects- against age-related degeneration [54]. Interestingly, many of these phytochemicals are activators of Nrf2 signaling and through this pathway they can inhibit ROS production and counteract oxidative damage [51]. Furthermore, hormetic phytochemicals have recently received considerable attention for their pro-longevity effects and for their ability to act as sirtuin activators [55]. Taking into account that the dietary habits of many centenarians seem to be extremely rich in phytochemicals, without neglecting the effect of genetic background, we hypothesize that long-lived people may have a constitutively upregulated Nrf2 pathway to respond better to cell stressors and thereby minimize cell damage. Moreover, considering that so-called "Blue Zones" that seem to harbor a high number of centenarians appear also to be areas with high nutrient density and low caloric density diets which may have led to a prolonged form of mild CR, the beneficial effects of their CR exposure may be also partially explained by a decrease in oxidative stress sensitivity, suggesting Nrf2 as a plausible effector of longevity signaling. Finally, it is important to consider that emerging evidence shows the close link between nutrient intake and inflammatory biomarkers [56]. It is well established that adipose tissue releases many inflammatory mediators and experimental studies have revealed the beneficial effects of CR in the attenuation of system-wide inflammatory processes [57]. Moreover, oxidative stress has been recognized to play a major role in determining and maintaining the low grade state of inflammation observed in ageing and age associated diseases [58]. Consistent with these statements, a number of studies have shown that the antioxidant-mediated Nrf2 activation is strongly associated with the protection from pro-inflammatory insults [59]. The activation of Nrf2 pathway might inhibit the production or expression of pro-inflammatory mediators including cytokines, chemokines, cell adhesion molecules, matrix metalloproteinases, cyclooxygenase-2 and inducible nitric oxide synthase [60]. Therefore, efficient inducers of Nrf2 activation, some of which are present in the diet of centenarians could be considered as effective means for the prevention of inflammation-mediated diseases.

## Conclusions

This review summarized some of the healthy ageing secrets of long-lived individuals. Presently it is of great interest to study characteristics of people living over far longer than the expected life span and to understand which factors are important in shaping longevity. The realization of healthy longevity is possible but to achieve a longer and a healthier life, increased attention must be placed on lifestyle choices, particularly the diet. There is a huge volume of scientific literature on diet and health but less attention has been paid to dietary patterns. Although it seems unlikely that there is a particular dietary pattern that promotes exceptional longevity, understanding the heterogeneity in dietary patterns and nutritional status of centenarians may provide a wealth of information relevant to human ageing. From a scientific perspective, a particular diet able to delay ageing may help to identify new molecules to extend and ameliorate age associated disease, opening new opportunities for drug discovery and companies working in nutrition and pharmacology. Furthermore, our knowledge of nutrient-sensing pathways has greatly increased in recent years and the modulation of these pathways by diet or pharmaceuticals can have a profound impact on health and thus represent a therapeutic opportunity for the extension of the human lifespan and quality of life improvement. In addition to the common pathways that regulate biological ageing there are also promising and attractive new targets for therapeutic interventions that can positively affect healthy ageing. The development of strategies that will lead to the extension of healthy life and that would result in slowing the rate of ageing and lowering risk for age associated disease may be part of the new paradigm for the biomedical sciences that can be termed 'positive biology'.

## Competing interests

The authors declare that they have no competing interests.

## Authors' contributions

SD, DCW, GS wrote the draft; all authors edited the paper and approved its final version.
